# Supplementary material for: Deconvolution volumetric additive manufacturing
Source: Nat Commun. 2023 Jul 21;14:4412. doi: 10.1038/s41467-023-39886-4 (PMC10362001; doi:10.1038/s41467-023-39886-4)
Supplement: Supplementary file 1 — Supplementary Information [file 41467_2023_39886_MOESM1_ESM.pdf]

Supplementary Information

Deconvolution volumetric additive manufacturing

Antony Orth[1]*, Daniel Webber[1]*, Yujie Zhang[1], Kathleen L. Sampson[1], Hendrick W. de Haan[2], Thomas Lacelle[1], Rene Lam[1], Daphene Solis[1], Shyamaleeswari Dayanandan[1], Taylor Waddell[3], Tasha Lewis[3], Hayden K. Taylor[3], Jonathan Boisvert[1], Chantal Paquet[1]

[1]National Research Council of Canada, Ottawa, Ontario, Canada

[2]Ontario Tech University, Oshawa, Ontario, Canada

[3]University of California Berkeley, Berkeley, California, USA

*These authors contributed equally to this work

Emails: antony.orth@nrc-cnrc.gc.ca; daniel.webber@nrc-cnrc.gc.ca

Supplementary Note 1. Measurement of optical PSF

To experimentally measure the intensity distribution of projected pixels in the resin, we performed experimental ray tracing using the fluorescence of the photoinitiator in the resin. For the VAM printer used in Figs. 1 – 6, a pattern of equally spaced, 1-pixel wide lines were projected by the projector through the resin. The resulting fluorescence traces the trajectory of the rays through the resin-filled vial, as shown in Figs. S1a-b. The images are recorded by the camera either positioned above the vial (Fig. S1a), or in the regular position (Fig. S1b) imaging through the side of the vial, perpendicular to the optical axis of the projector. In each case, the central-most line travelling through the vial is identified

by a white dashed box. The width of the dashed box is 10 mm, matching the diameter of the disks from Figs. 1-4. The PSF for each orientation is then obtained by averaging the fluorescence intensity recorded along the length of each line within the dashed box. The resulting experimentally determined average PSFs are shown in Figs. S1c-d (blue curves). Gaussian curves are fit to these PSFs to extract the full width at half-maximum (FWHM) for each orientation: $0.190\ (\pm 0.004)\mathrm{mm}$ for PSF_z and $0.120\ (\pm 0.004)\mathrm{mm}$ PSF_{xy}. The same procedure was applied to the printer used in Fig. 7, and the FWHM values for each orientation were: $0.101\ (\pm 0.004)\mathrm{mm}$ for PSF_z and $0.0800\ (\pm 0.004)\mathrm{mm}$ PSF_{xy}.

Measuring the PSF of the printer is challenging because we cannot physically put a detector into the resin itself to measure the light intensity distribution from a pixel. Imaging the fluorescence of projected beams is a convenient way to make this measurement, however there is an important consideration with respect to the imaging depth of field. Most importantly, there is not enough signal to image the fluorescence intensity from a single projected pixel. Therefore, a line of pixels must be projected. While this increases the signal, the camera records out-of-focus blur, which increases the apparent width of the line. We counteract this effect by stopping down the aperture of the imaging camera until we reach a compromise between a large imaging depth of field (reduced out of focus blur) while still collecting enough signal to make a good measurement. This is further mitigated by the Gaussian fits in Figs. S1c-d, which ignores the long tails caused by the out of focus blur. As a result, we expect that the Gaussian fits report a good metric for the true width of a projected pixel.

Supplementary Note 2. Microgravity print

The diffusive component of dose spread is faster in lower viscosity resins, potentially making deconvolution correction even more critical. We tested the effectiveness of dose diffusion correction in low-viscosity PEGDA700 ($\mu = 100\text{cp}$) by printing functional parts in a micro-gravity environment. By printing the parts in micro-gravity we avoid the undesirable effects of part buoyancy (i.e. sedimentation, floating), which also degrades print quality in low viscosity resins. Micro-gravity was achieved by printing using the spaceCAL system [1] on-board an aircraft performing a sequence of parabolic altitude maneuvers (see next Section for printer details). Fig. S12a shows the reference design of a ¼-20 bolt and corresponding nut [2]. These parts were printed with and without deconvolution correction. To enable printing within the 20 second microgravity window, the projection intensity was increased by scaling up the pixel values, leading to a small amount of saturation of projector pixels (see Methods – Print Calculation). Parts printed on Earth without deconvolution correction have the correct gross shape, but the threads do not form (Fig. S12b). On-Earth deconvolution correction yields visible threads, but with minimal groove depth. During printing, it was observed that the partially formed bolt would float, resulting in non-ideal dose delivery. In contrast, parts printed in micro-gravity retained their threads as shown in S12d,e. The high fidelity of the deconvolution-corrected microgravity prints enables these parts to be used for their intended purpose, as demonstrated by screwing the nut onto the bolt (Fig. S12f). Although threads are visible on the uncorrected microgravity print, their shape is rounder and their depth (0.32 mm, Fig. S12g) is shallower than for the deconvolution-corrected print (0.49 mm, Fig. S12h). We attribute these differences to the large diffusive dose spread expected for a 100 cp resin, in addition to projector optics, both of which are mitigated by our deconvolution correction approach.

Supplementary Methods. Microgravity printer details (SpaceCAL)

The SpaceCAL system was designed to incorporate five individual VAM systems to allow simultaneous fabrication in five vials. Vials were stacked on each other for a total of 5 vials and conjoined into a vial stack. This design enabled 25 printing experiments to be conducted before any manual intervention from the user was required. The projectors were attached to a carriage that was able to move vertically between printing operations to address each vial in the stack. Wintech 4710pro projectors were used with an illumination wavelength of 405nm, maximum intensity at the image plane of 45 mW/cm^2, and image projection distance of 157mm. The projectors had a spatial resolution of 1080p with a pixel size of 52 µm at the focal plane within the photopolymer. The intensity at each pixel was controlled with 8-bit resolution at a frame rate of 60 Hz. The vials used were borosilicate glass with 32 mm outer diameter and 1.4 mm wall thickness and were cut to 64 mm in length. The vials were rotated at 54deg/s during printing. For this experiment a ZeroG aircraft executed 30 parabolas to simulate various gravitational conditions. The duration of these microgravity gravitational conditions within each parabola flown was between 20 and 28 seconds.

Supplementary Note 3. Experiment design

1. **Disk polymerization time study (Figs. 1,3,4,S4,S5,S7,S8)**

   Independent variables:

   - Resin viscosity (via resin type): DUDMA, $\mu = 8{,}645\ \mathrm{cP}$ and DUDMA/PEGDA, $\mu = 1{,}750\ \mathrm{cP}$.
   - Illumination intensity: 4.9 mW/cm^2, high power: 9.8 mW/cm^2. These values correspond to the intensity at a pixel set to the maximum gray value (255) of the 8-bit projector.
   - Disk thickness (h) = [1.07 , 0.91, 0.80, 0.70, 0.59, 0.48, 0.37, 0.27, 0.16, 0.05]mm for 4.9 mW/cm^2 illumination; [0.85, 0.53 , 0.43, 0.43, 0.37, 0.27, 0.21, 0.16, 0.11, 0.05]mm for 9.8 mW/cm^2 illumination. Thinner disks were used for higher power due to the reduced effect of diffusion.

   Response variable:

   - Polymerization time (τ_p).

   Measurement uncertainty of response variable:

   - Standard deviation of triplicate experiments (Figs. S5,S7,S8).

2. **Diffusion coefficient experiment (Fig. 2)**

   Constant parameters:

   - Thickness of thin disk $(d_0 = 0.5\ \mathrm{mm})$.

- Thickness of thick disk ($d_1 = 5 \text{ mm}$).
- Initial exposure time (t_{app}) = 40s.
- Projector illumination intensity (4.9 mW/cm^2)

Independent variables:

- wait time (t_w) = [0, 107, 149,234,429,2046]s for DUDMA/PEGDA; (t_w) = [0, 166, 259,559,1270,2475]s for DUDMA.
- Resin viscosity (via resin type): DUDMA, $\mu = 8{,}645 \text{ cP}$ and DUDMA/PEGDA, $\mu = 1{,}750 \text{ cP}$.

Response variables:

- Time to polymerization of thin disk ($T_{ex}(t_w, \mu)$) with wait time.
- Polymerization threshold exposure time ($t_{th}(\mu)$) with no wait time. Used for model fitting.

Measurement uncertainty of response variables:

- $T_{ex}(t_w, \mu)$ and $t_{th}(\mu)$ are identified as when optical scattering crosses a threshold value gray value G = 6 +/- 1 gray levels above the background intensity level of the unpolymerized resin. The value G=6 is chosen so that it is well above the background level of the camera (~18 gray levels), and well above the noise floor (1 gray level). Ideally, in the absence of camera noise, we would identify polymerization onset as an infinitesimal increase in the scattering intensity above the background. However, in a real experiment, we must raise this

threshold so that it is separated from the background. The effect of choosing a different G values (+/- 1) is reported via error bars in Fig. 2b.

Dependent variables:

- $t_{delay} = t_w + T_{ex} + t_{app}/2$

Model fitting inputs

- $t_{delay}, T_{ex}, t_{app}, h_0, t_{th}$
- The model is based on Eq. 2. Note that the D depends on resin type via μ, but the model does not incorporate the effect of μ on D. Therefore, we fit the model in Eq. 2 independently for each of the two resins used.

Model fitting outputs

- D (in units of mm^2/s).
    - o Uncertainty quantified by resulting fit value of D when different gray values for G (=6 +/- 1) are used for measurement of t_{th}, T_{ex}. These upper and lower bound fits are shown as dashed curves in Fig. 2b.

Supplementary Note 4. Derivation of Eq. 2

Below we derive a simple 1D model to extract a dose diffusion coefficient from the experiment in Fig. 2a. During the initial exposure of the thin disk, the applied dose is assumed to be a square pulse in the z-direction and uniform in time:

$$D_{init}(z,t) = \begin{cases} I_0 t, & 0 < t < t_{app} \text{ and } -h_0/2 < z < h_0/2 \\ 0, & \text{elsewhere} \end{cases} \tag{S1}$$

Where I_0 is the light intensity, assumed to be uniform within the projected disk region. During this exposure, the applied dose is continuously diffusing as more dose is accumulated. For the sake of simplicity, we approximate the dose distribution by what would have been obtained by an instantaneous exposure at time $t_{app}/2$. Under this approximation, the diffused dose at the center of the disk is:

$$D_{init}\left(z=0, t_{app}\right) = I_0 t_{app} \int_{-h_0/2}^{h_0/2} H\left(z, t_{app}/2\right) dz = I_0 t_{app} \times \operatorname{erf}\left(\frac{h_0}{4\sqrt{D t_{app}/2}}\right) \tag{S2}$$

Where D is the diffusion coefficient, and H is the 1D diffusion kernel:

$$H(z,t) = \frac{1}{\sqrt{4\pi D t}} \exp(-z^2/4Dt) \tag{S3}$$

Similarly, after the wait period, during which $D_{app}(t) = 0$, we have

$$D_{init}\left(t_{app} + t_w\right) = I_0 t_{app} \int_{-h_0/2}^{h_0/2} H\left(z, t_w + t_{app}/2\right) dz = I_0 t_{app} \times \operatorname{erf}\left(\frac{h_0}{4\sqrt{D \times \left(t_w + \frac{t_{app}}{2}\right)}}\right) \tag{S4}$$

During the second "extra" exposure, the entire region explored by dose diffusion from the thin disk is exposed uniformly (this is the thick disk region, which is 10x thicker than the thin disk). Thus, within the thin disk region, the dose is uniformly elevated during this exposure. Therefore, we only need to consider further diffusion of the dose from the initial exposure. We assume that this initial dose continues to diffuse with the same diffusion coefficient until the onset of polymerization is observed via scattering imaging. The duration of the second exposure is T_{ex} and the extra dose applied in this second exposure is D_{ex}.

To relate the polymerization dose threshold D_{th} to the dose imparted from the initial (D_{init}) and extra (D_{ex}) exposures, we use the linearity of the diffusion equation:

$$D_{th} = I_0 t_{th} = D_{init}\left(t_{delay}\right) + D_{ex}(T_{ex}) \tag{S5}$$

In terms of experimental parameters, these doses are expressed as:

$$D_{init}(t_{delay}) = I_0 t_{app} \times \text{erf}\left(\frac{h_0}{4\sqrt{D t_{delay}}}\right) \tag{S6}$$

$$D_{ex}(T_{ex}) = I_0 T_{ex} \tag{S7}$$

Where $t_{delay} \equiv t_w + \frac{t_{app}}{2} + T_{ex}$. Finally, we arrive at an equation linking the threshold dose to the experimentally measured delay time:

$$D_{th} = I_0 t_{th} = I_0 T_{ex} + I_0 t_{app} \times \text{erf}\left(\frac{h_0}{4\sqrt{D t_{delay}}}\right) \tag{S8}$$

Which can be rearranged to:

$$T_{ex} = t_{th} - t_{app} \times \text{erf}\left(\frac{h_0}{4\sqrt{D t_{delay}}}\right) \tag{S9}$$

This form of the diffusion model is convenient because the threshold exposure time t_{th} can measured to high accuracy by recording the time to polymerize when there is no wait period. This equation is then fit to the experimental data in Fig. 2b to obtain the diffusion coefficients D reported for each resin.

Supplementary Note 5. Example Python code (Supplementary Code 1)

The attached Python code provides an example implementation of target geometry deconvolution. Executing the python script takes an input 3D voxel array (gyroid.npy), and outputs a corrected target geometry. This geometry can then be used as the target geometry for projection computation in VAM.

Supplementary Figures

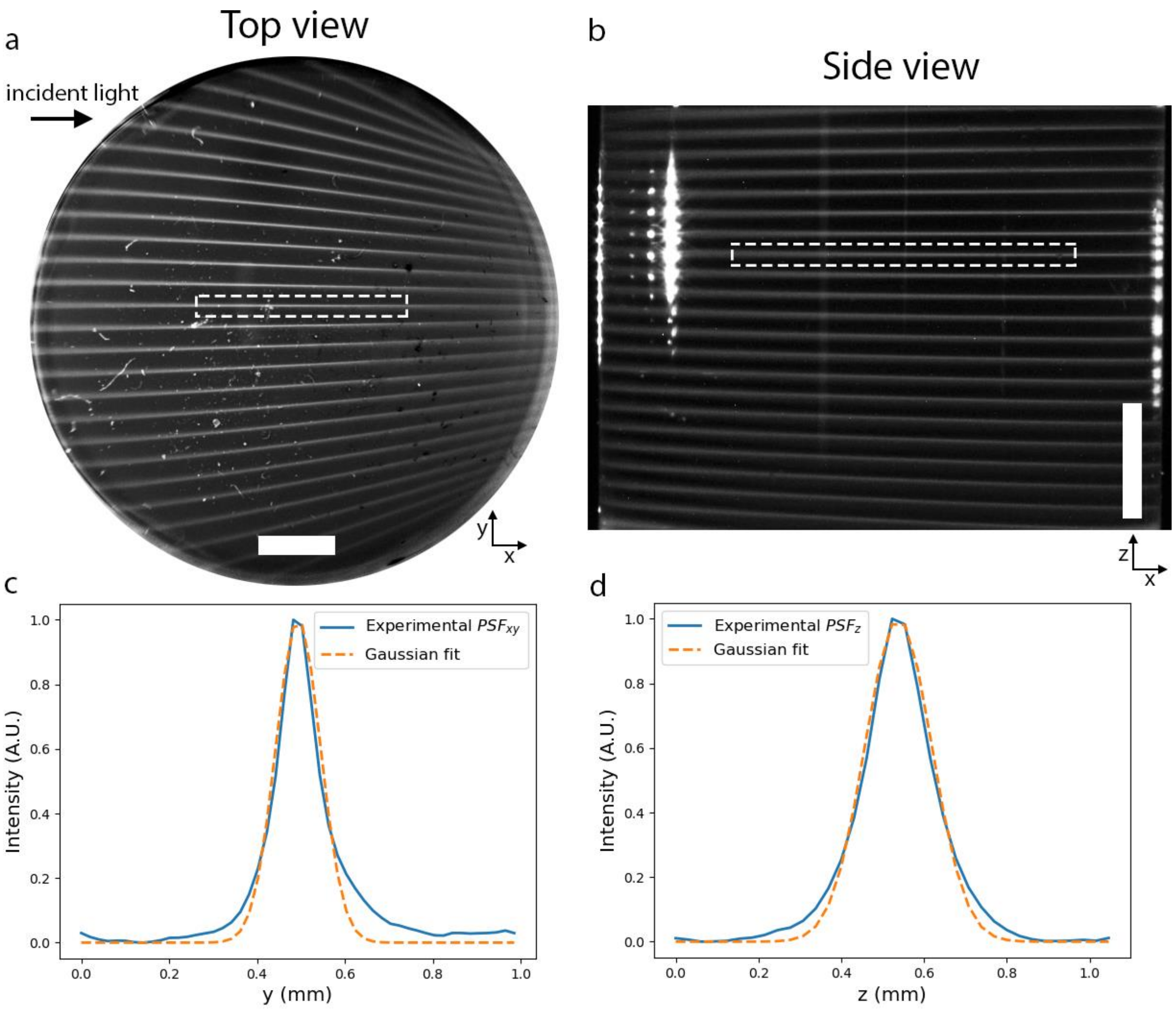

Fig. S1. **Measurement of projector PSF in vial**. a) Top view fluorescence image resulting from projecting a series of 1-pixel wide lines through the resin filled vial. Light propagation is from left to right. b) Same as in (a) but for the side view. The dashed boxes in (a) and (b) are the analysis regions for calculating the experimental PSFs in (c) and (d), respectively. Scalebars are 5mm. c) – d) Experimental PSFs (solid blue curves) and Gaussian fits to the experimental PSFs (dashed orange curves) for in-plane (xy) and vertical (z) directions, respectively.

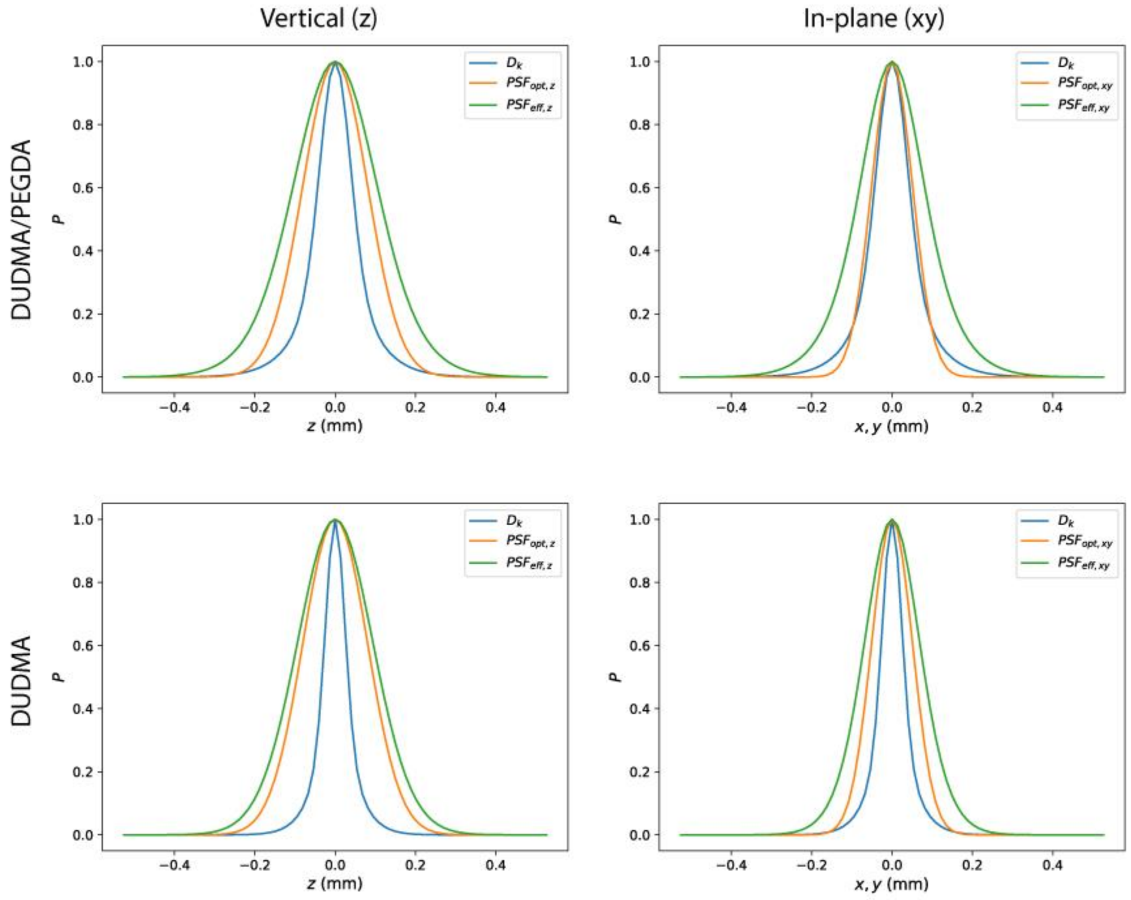

Fig. S2. **Diffusion kernels, optical PSFs and effective PSFs**. Vertical and in-plane directions are left and right columns, respectively. Top/bottom rows are DUDMA/PEGDA and DUDMA resins, respectively.

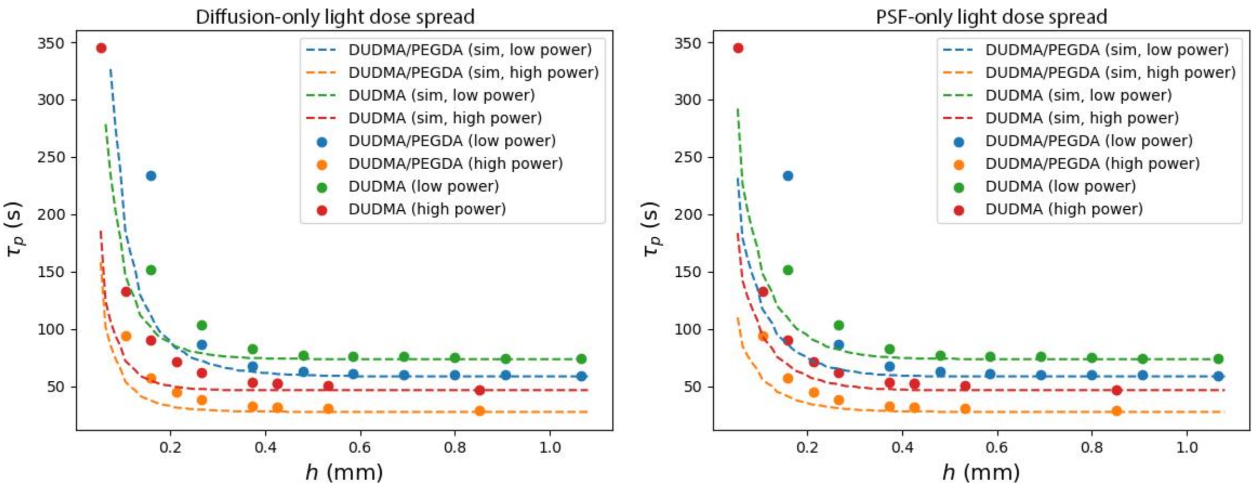

Fig. S3. **Light dose spread due to projector PSF and diffusion only in isolation do not explain the observed experimental data**. Polymerization time simulations with light dose spread modeled as only due to diffusion (left), or only due to the projector's optical PSF (right). Neither model captures the correct behaviour, whereas the combined model from Fig. 3 does. Each datapoint is an individual experimental measurement. Datapoints of the same color are all obtained from a single print.

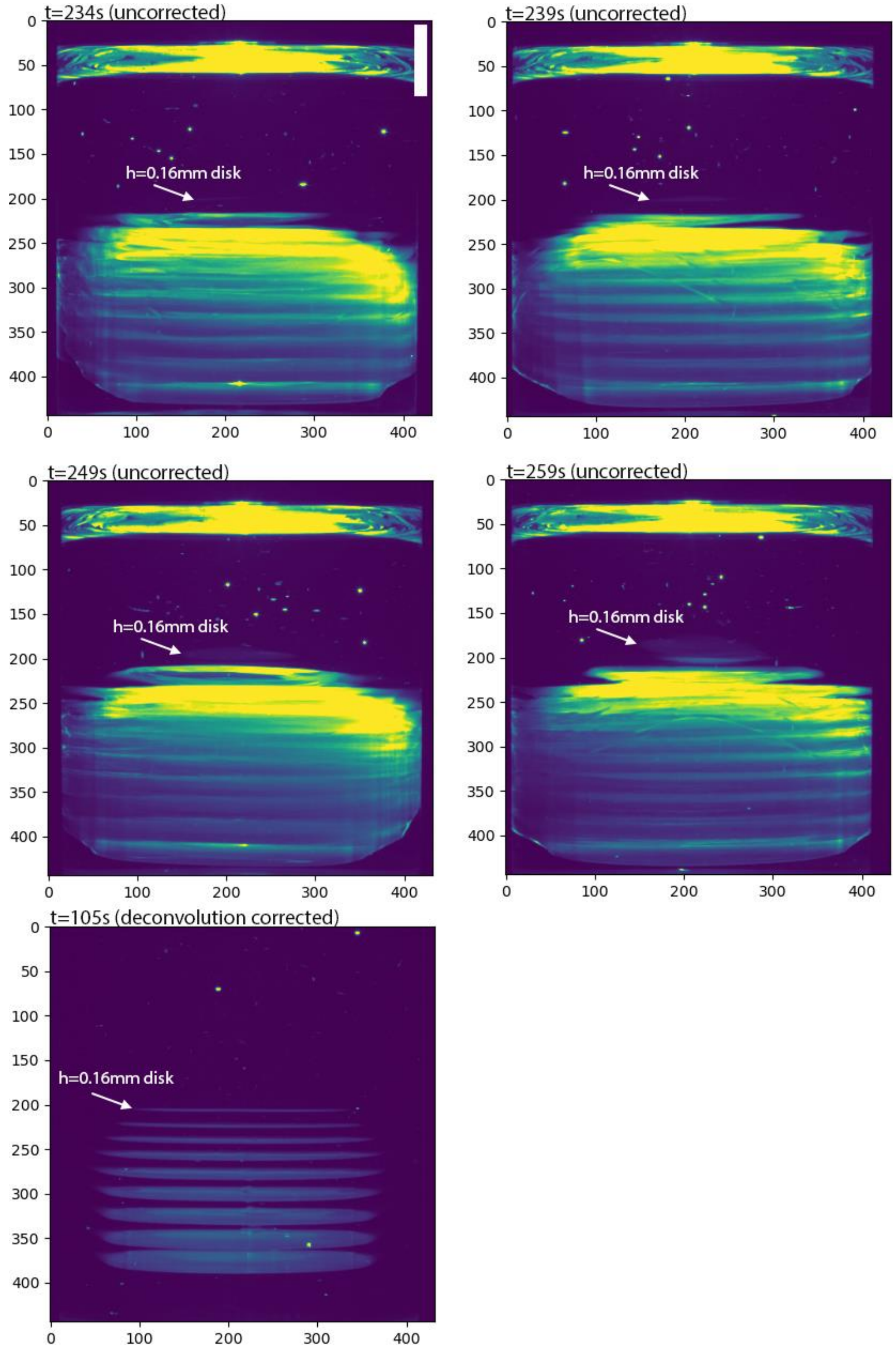

Fig. S4. Possible thermal effects compromise thin disk formation in DUDMA/PEGDA resin without deconvolution correction. Raw scattering intensity images captured by the camera during the disk experiment shown in Figs. 1,3,4a-b (DUDMA/PEGDA, low power). The $h = 0.16mm$ disk begins to polymerize at $t \approx 234s$, but soon starts to become deformed, tilting and

travelling upwards. The disk never fully forms, likely due to the influence of convection from the heat generated by polymerization of the other disks. This datapoint is included in Fig. 3 with an asterisk to indicate that the polymerization time deviates significantly from that predicted by the model, which does not include the effects of convective flow. The bottom left figure shows an image frame from a deconvolution corrected print in the same resin, where the $h = 0.16mm$ forms properly. Moreover, the thicker disks are only slightly overcured, compared with the uncorrected prints where the disks are overcured to the vial wall, despite the design diameter of 10mm (vial diameter 24.8mm).

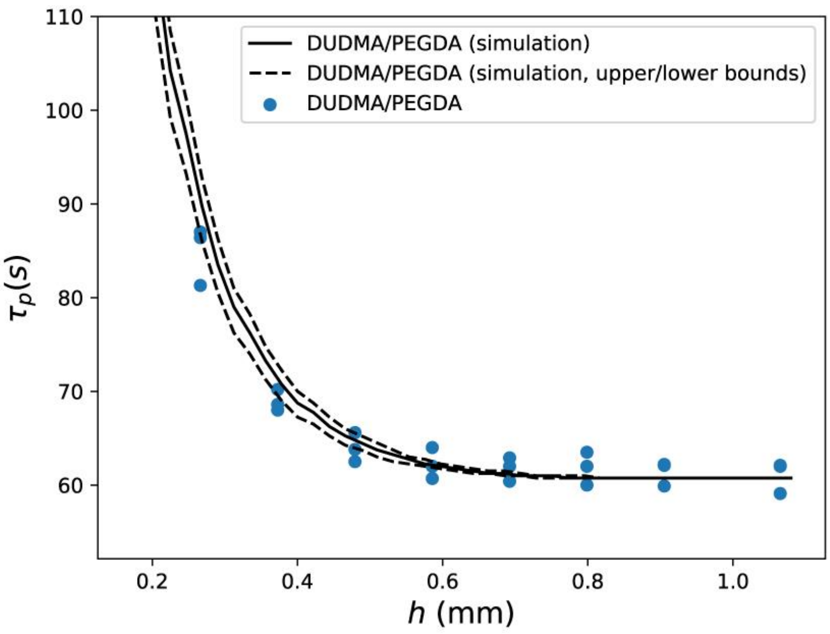

Fig. S5. **Polymerization time as a function of disk thickness for 3 replicates of DUDMA/PEGDA resin**. The curves are simulations as described in the “Combined diffusion and optical PSF model” section of the main text. The upper/lower bounds correspond to simulations with the diffusion coefficients and optical FWHMs set to one standard deviation above/below the best fit values, as reported in the main text. Each datapoint is an individual experimental measurement.

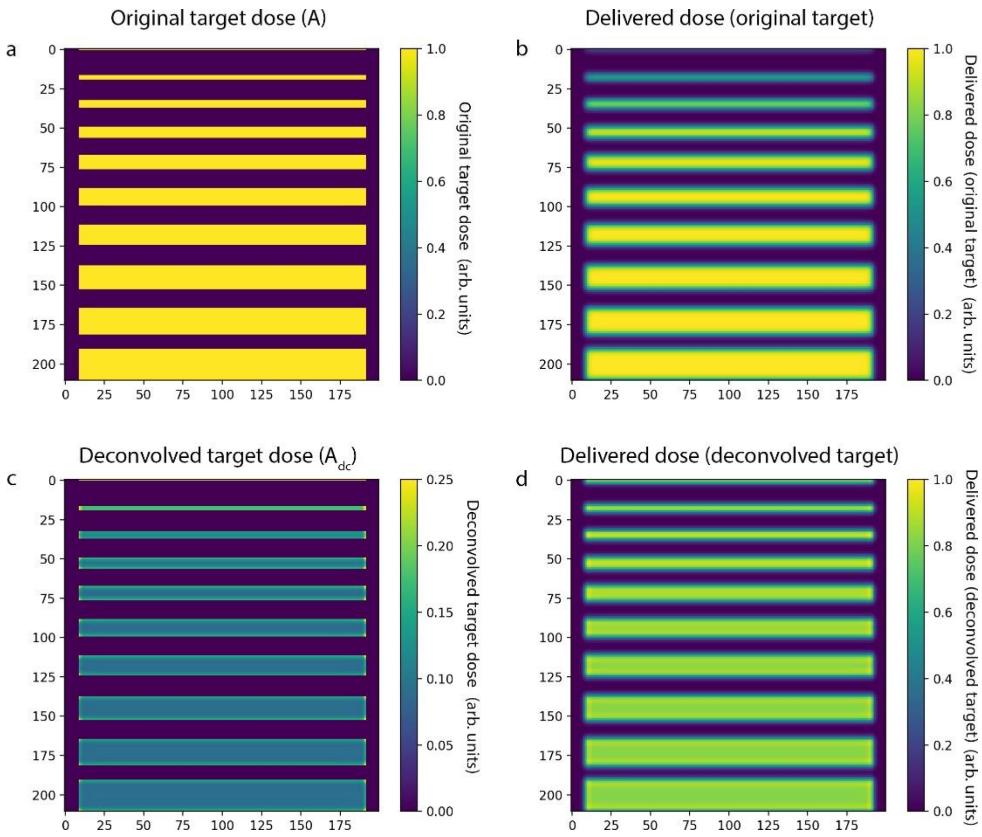

Fig. S6. **Target disk geometries used for calculating tomographic projections**. a) Original target dose. b) Simulated delivered dose of the original target geometry, taking into account dose diffusion and the optical PSF. c) and d) As for (a)-(b), but for the deconvolved target dose (number of iterations $n_i = 10$). Note the increased target dose near the edges of the disks in (c).

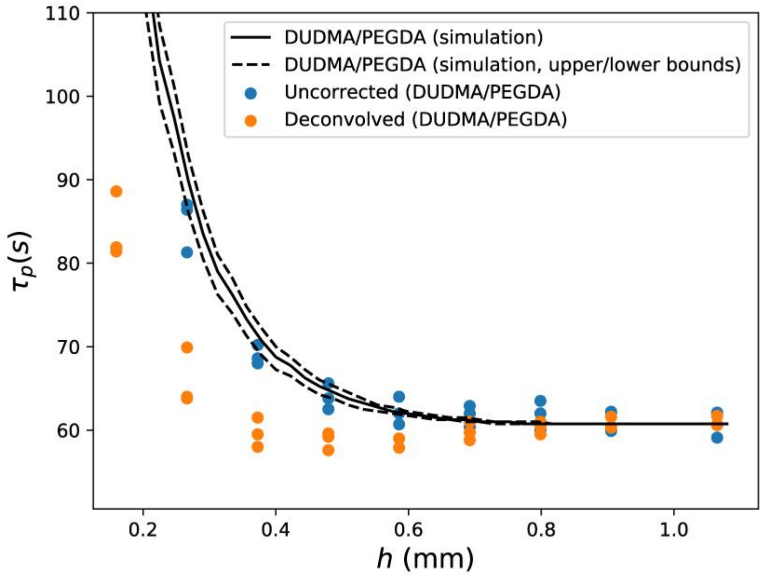

Fig. S7. **Effect of deconvolution on disk geometry: triplicate experiment data**. The same plot as in Fig. S5, with deconvolution-corrected datapoints (3 replicates) shown in orange. Each datapoint is an individual experimental measurement.

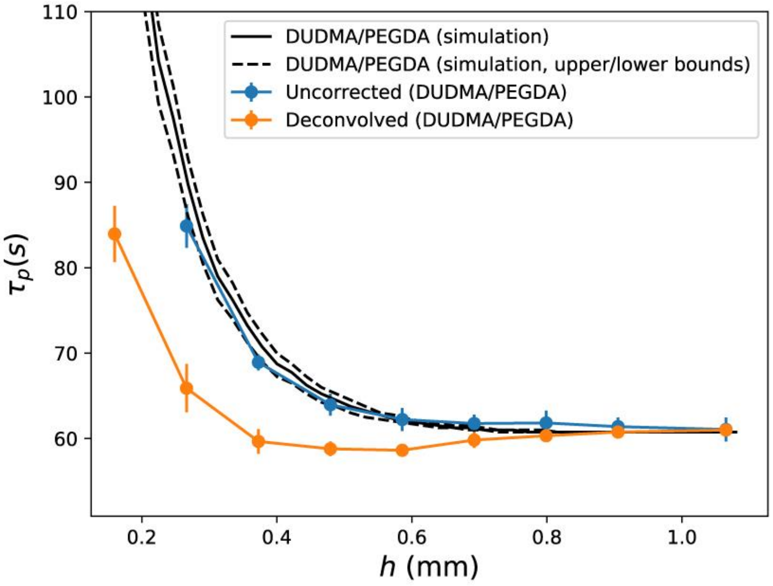

Fig. S8. **Effect of deconvolution on disk geometry: triplicate experiment data.** The same plot as in Fig. S7, with replicate datapoints replaced with error bars corresponding to one standard deviation from the mean. Deconvolution correction significantly improves simultaneity of print times beyond the experimental variation for the uncorrected print.

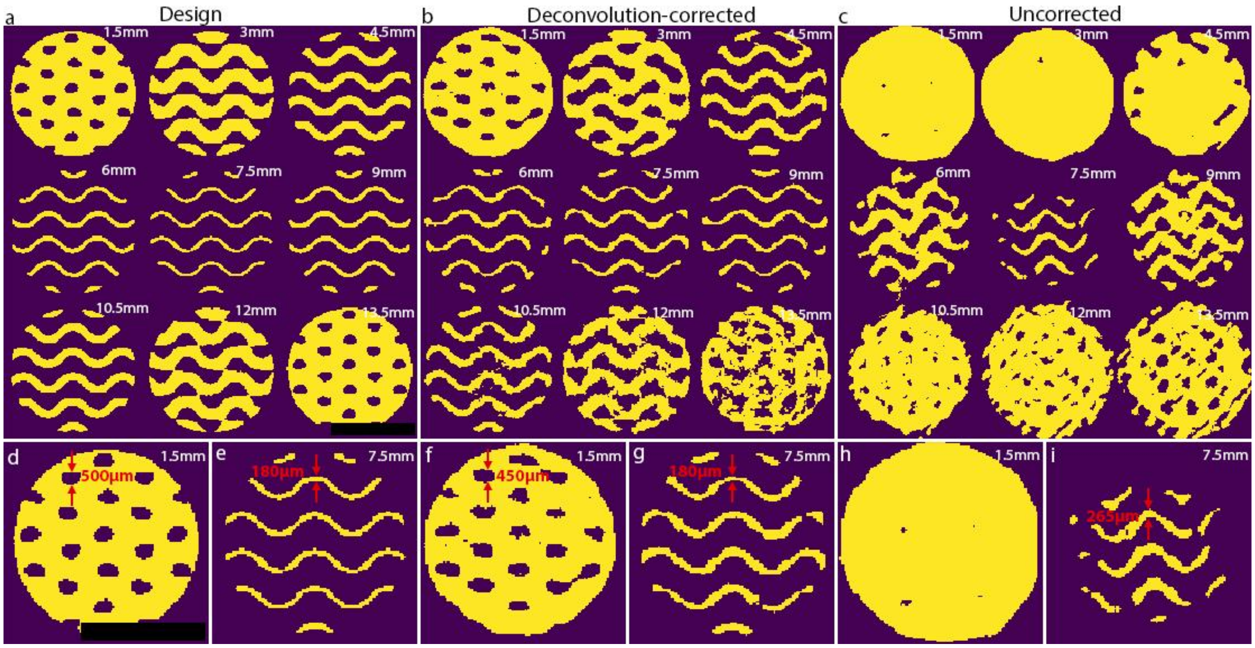

Fig. S9. Visualization of the print geometry of a complex part. a)-c) 2D sections of the variable wall thickness gyroid. The distance of the section from the top surface of the gyroid is noted in the top right of each section. Yellow denotes inside the object, and blue denotes outside the object. For (b) and (c), sections are obtained from OST reconstructions. d),e) Magnified views of top and middle sections, respectively, showing the differing geometries. The top section (d) is characterized by an array of pores of diameter $\approx 500\mu m$. The middle section is characterized by wavy walls of thickness $\approx 180\mu m$. f), g) Equivalent sections as in (d) and (e), for the deconvolution-corrected print. (h) and (i), as in (d) and (e), but for the uncorrected print. Pores are overcured in the top section and therefore are not annotated in (h).

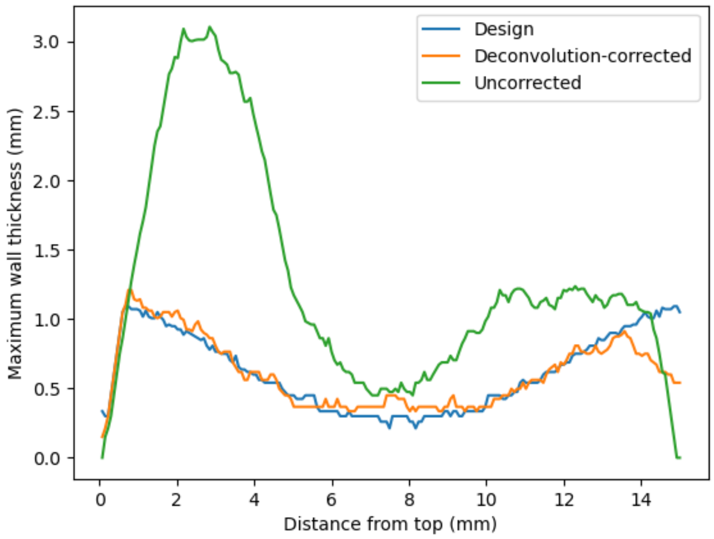

Fig. S10. **Maximum wall thickness of variable wall thickness gyroid, as measured by the Euclidean distance transform**. The design is an approximately sinusoidally varying wall thickness from top to bottom. The deconvolution-corrected print matches the designed variation in maximum wall thickness, whereas the uncorrected print drastically overshoots the designed wall thickness at the top and bottom of the gyroid. For the walls in the middle (~7.5mm from the top), wall thickness can vary due to voxelization and meshing. The maximum wall thickness is reported here due to computational simplicity, however, the minimum wall thickness is shown in Fig. S9e and reported in the main text.

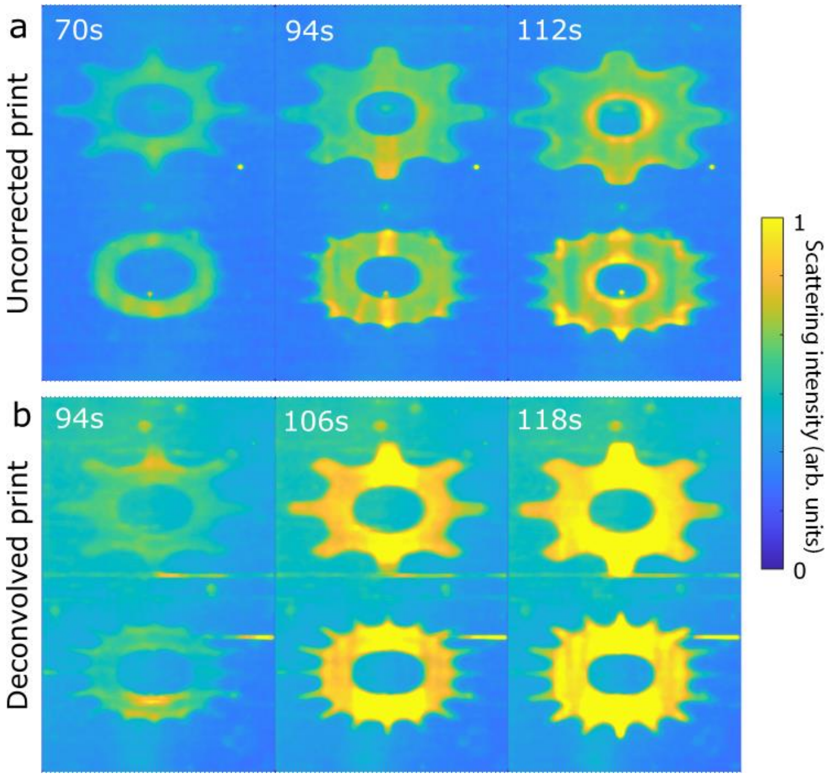

Fig. S11. Gears with fine teeth are printable in VAM with deconvolution correction. Optical scattering images at different times during printing for gears printed a) without correction and b) with deconvolution correction. The parts appear horizontally-elongated due to lensing caused by the cylindrical vial. Horizontal line artifacts in b) represent scattered light from scratches in the glass vial.

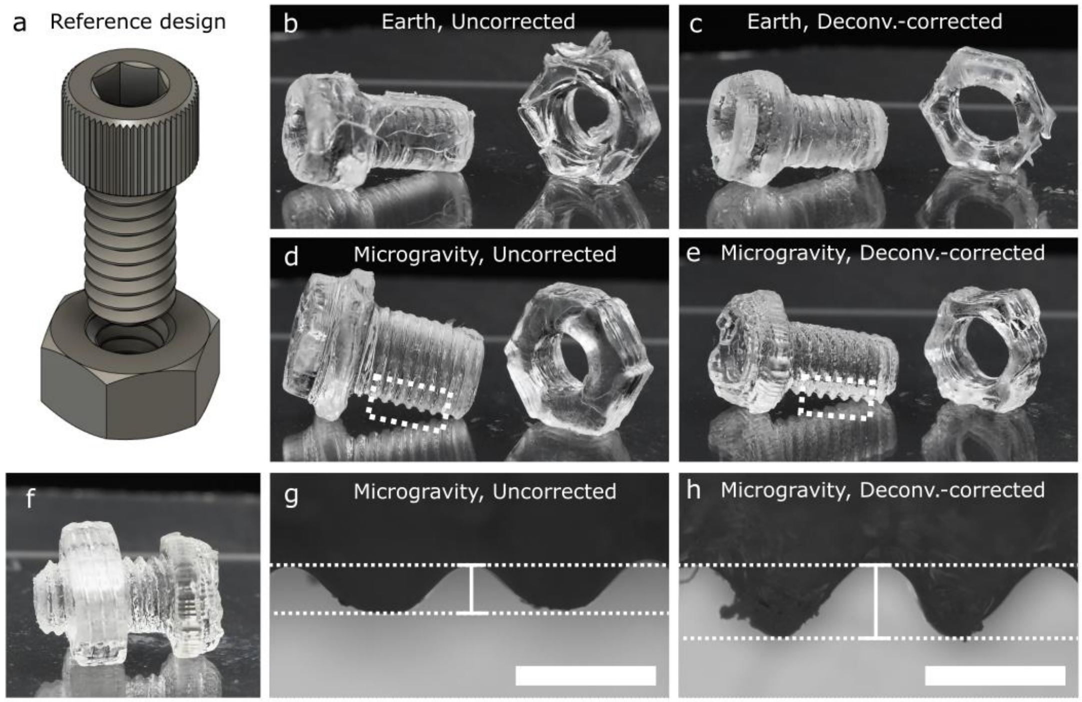

Fig. S12. **Deconvolution correction may improve print fidelity for low viscosity resin**. a) Isomorphic view of the reference design of a ¼-20 nut and bolt. Below is a plane view of the threads on the bolt in the reference design. STL files for these two parts is obtained from the McMaster-Carr website[1,2]. b) Reference design printed on-Earth without dose deconvolution-correction. c) Same as b) but with dose deconvolution correction. d) Same as (b) but in microgravity. d) Same as (c) but in microgravity. f) Photograph showing the nut screwed onto the bolt for the deconvolution-corrected microgravity print. g,h) Microscope images of the threads on the printed bolts shown in (d) and (e) respectively. All scalebars are 1 mm.

Supplementary Tables

	Large gear, uncorrected	Large gear, deconvolution-correction	Small gear, uncorrected	Small gear, deconvolution-correction
Mean tooth width (mm)	0.523 [0.4]	0.387 [0.4]	0.161 [0.1]	0.127 [0.1]
Mean tooth length (mm)	0.479 [0.5]	0.484 [0.5]	0.198 [0.5]	0.383 [0.5]
RMS error: tooth width (mm)	0.141	**0.042**	0.070	**0.040**
RMS error: tooth length (mm)	0.051	**0.041**	0.306	**0.124**

Table S1. Gear teeth geometry for prints in Fig. 7. The numbers in square brackets denote the design values. For both small and large-toothed gears, the RMS error for tooth width and length are lower for the deconvolution-corrected print.

	**Resin**	**Max illumination intensity**	**Response variable / print fidelity metric**	**Purpose**	**Figure #**
**Stack of disks**	DUDMA/PEGDA, DUDMA	4.9, 9.8 mW/cm^2	Time to polymerization vs. disk thickness. 3 replicates in Figs.S5,S7,S8.	To demonstrate large variation of polymerization time as a function of disk thickness.	1,3,4,S4,S5,S7,S8
**Two-stage disk print**	DUDMA/PEGDA, DUDMA	4.9 mW/cm^2	Time to polymerization vs. wait time between print stages	To determine dose diffusion coefficient via fit.	2
**Variable wall thickness gyroid**	DUDMA/PEGDA	4.9 mW/cm^2	Jaccard index, maximum wall thickness along height (Figs. S9,S10)	To demonstrate correction for non-trivial 3D geometry.	5,6,S9,S10
**Gears**	DUDMA/PEGDA	20 mW/cm^2	Jaccard index, Tooth length and width (Table S1).	To demonstrate correction for non-trivial geometry.	7,S11
**Nut and Bolt**	PEGDA	45 mW/cm^2	Thread depth.	To demonstrate deconvolution correction for low viscosity resin.	S12

Table S2. Summary of prints performed in this paper.

Supplementary References

1. https://www.mcmaster.com/92196A537/
2. https://www.mcmaster.com/95462A029/
