# Supplementary material for: Deconvolution volumetric additive manufacturing
Source: Nat Commun. 2023 Jul 21;14:4412. doi: 10.1038/s41467-023-39886-4 (PMC10362001; doi:10.1038/s41467-023-39886-4)
Supplement: Supplementary file 3 — Description of Additional Supplementary Files [file 41467_2023_39886_MOESM3_ESM.pdf]

Description of Additional Supplementary Files

File Name: Supplementary Code 1
Description: Example Python code. The attached Python code provides an example implementation of target geometry deconvolution. Executing the python script takes an input 3D voxel array (gyroid.npy), and outputs a corrected target geometry. This geometry can then be used as the target geometry for projection computation in VAM.
